# Lighting up the muscles: ^18^F-FDG PET/CT in rare disseminated tuberculous myositis

**DOI:** 10.22038/aojnmb.2025.90177.1658

**Published:** 2026

**Authors:** K Vidhya Kannappan, Vivek Kumar Saini, Vandana Kumar Dhingra, Venkatesh S Pai, Sryla Punjadath

**Affiliations:** 1 Department of Nuclear Medicine, AIIMS Rishikesh, Uttarakhand, India; 2 Department of Medicine & Rheumatology, AIIMS Rishikesh, Uttarakhand, India; 3 Department of Medicine, AIIMS, Rishikesh, Uttarakhand, India

**Keywords:** PET/CT, Extrapulmonary tuberculosis Musculoskeletal TB Disseminated extrapulmonary TB, Mycobacterium tuberculosis A B S T R A C T

## Abstract

Tuberculous pyomyositis is a rare and often under-recognized extrapulmonary manifestation of tuberculosis, presenting with non-specific symptoms such as fever and abscess, that may delay diagnosis. We report a unique case of disseminated tuberculous pyomyositis in a 56-year-old male with underlying chronic inflammatory arthritis. The diagnosis was confirmed using CBNAAT, line probe assay (LPA), and histopathology. Currently MRI is the standard imaging modality in use for imaging pyomyositis. ^18^F-FDG PET/CT can play a crucial role in detecting widespread metabolically active lesions involving multiple skeletal muscle groups and lymph nodes, which are not fully appreciated on conventional imaging and prove to be a sensitive imaging modality in such cases. This imaging modality provided a comprehensive assessment of disease burden, helping to map the full extent of involvement and guide clinical management. Early identification and treatment are essential, especially in atypical or disseminated cases, to prevent further complications and improve patient outcomes. Here we present a rare case presentation of such extensive muscular involvement in tuberculosis, highlighting the diagnostic utility of PET/CT in extrapulmonary TB.

## Introduction

 Tuberculosis (TB) remains a major global health burden, with extrapulmonary TB (EPTB) constituting approximately 15–20% of all TB cases, with higher prevalence in immuno-compromised populations (2). 

 Musculoskeletal TB accounts for only 1–3% of EPTB, and involvement of skeletal muscle is extremely rare due to its inherent resistance to Mycobacterium tuberculosis infection, attributed to high vascularity, lactic acid content, and lack of reticuloendothelial tissue (3, 5, 6). Tuberculous pyomyositis, characterized by suppurative infection of skeletal muscle, is therefore an unusual clinical entity and is often underdiagnosed due to its nonspecific presentation. Disseminated forms, with multi-muscle group involvement, are exceptionally rare, with a reported prevalence of around 1.8% in selected cohorts (5), and are typically seen in patients with significant immune suppression (1, 3, 7).

 Currently magnetic resonance imaging (MRI) is the standard imaging modality available in diagnosis of pyomyositis with high sensitivity and specificity. Recent advances in hybrid imaging, particularly ^18^F-fluorodeoxyglucose positron emission tomography/computed tomography (^18^F-FDG PET/CT), have enhanced diagnostic accuracy by enabling whole-body disease mapping, identification of metabolically active lesions, and targeted biopsy planning (4, 8–10). Early integration of PET/CT can improve detection, guide management, and assess treatment response in musculoskeletal TB. 

 Here, we present a rare presentation of disseminated tuberculous pyomyositis with extensive multi-muscle group involvement detected on ^18^F-FDG PET/CT in an immunocompetent patient.

## Case report

 Tuberculous pyomyositis is a rare and often underdiagnosed manifestation of extra pulmonary tuberculosis. A 56-year-old male with a known history of chronic inflammatory small joint polyarthritis for the past 3 years presented with complaints of progressively increasing painful subcutaneous swellings and abscesses over the left thoracic & lower back region with multiple ulcerating nodular lesions over the right forearm, palm, right thigh, and left elbow, associated with progressive proximal muscle weakness, low-grade fever with chills, and significant weight loss. CBNAAT testing of pus aspirated from an abscess was positive for Mycobacterium tuberculosis, sensitive to rifampicin. A skin biopsy showed non-necrotizing granulomatous inflammation. LPA from an abscess confirmed rifampicin-sensitive Mycobacterium tuberculosis, and HRZE therapy was initiated. Patient was not on steroids or other immunosuppressive drugs (on ATT for disseminated TB). 

 Significant laboratory parameters- CBC: Hb: 8.3 g/dL; TLC: 15000cells/uL; DLC (N/L/M): 87/5/7; Platelets: 272000/ uL; ESR: 95 mm/hr (0-15); LDH: 95 U/L(0-247U/l).

**Figure 1:**
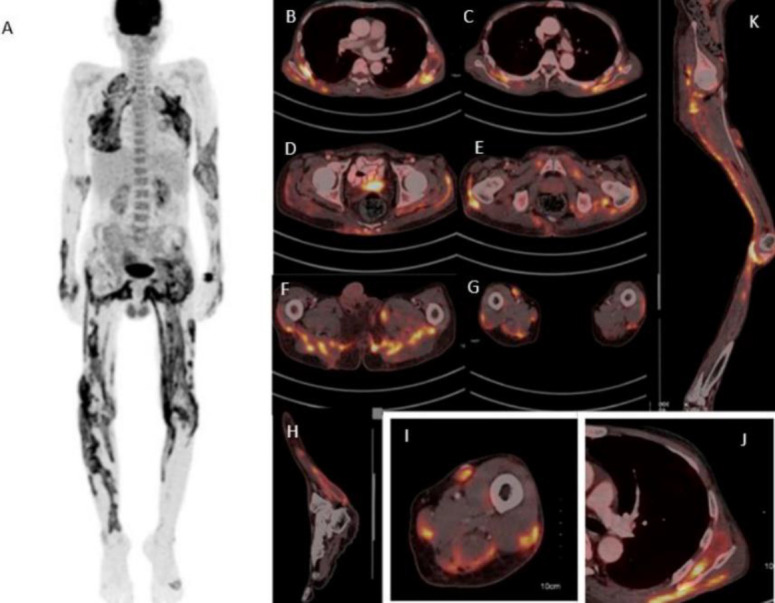
^18^F-FDG PET/CT demonstrated extensive metabolically active linear areas of FDG avidity across multiple muscle groups including parascapular, chest wall, gluteal, thigh, and leg muscles, with associated intra- and intermuscular hypodense collections, active subcutaneous nodular deposits in bilateral thigh region & abdominal, mesenteric and inguinal lymphadenopathy- consistent with disseminated tuberculous pyomyositis with extensive muscular, subcutaneous, and nodal involvement

 To better delineate the disease burden and activity, ^18^F-FDG PET/CT) was performed which revealed disseminated tuberculous pyomyositis involving multiple muscle groups (Figure 1). 

 PET scan was advised in this scenario for assessment of disease extent, the stage of disease and activity to guide therapy along with sites to go for biopsy/ pus cultures- as the patient was on regular ATT but not responding or showing signs of improvement. To the best of the author’s knowledge, this represents a rare presentation of disseminated TB pyomyositis involving various muscles groups throughout the body.

 During the course of hospitalization, patient was on ATT and also started on prophylactic antibiotics, following which pus cultures, aerobic cultures, KOH mount and modified ZN staining for nocardia were all found to be negative. Pus culture was positive for mycobacterium tuberculosis with no rifampicin resistance detected. 

## Discussion

 Tuberculosis is one of the endemic diseases in India with incidence of 210 per 100000 population as per WHO global TB health report, 2022. EPTB refers to any bacteriologically confirmed or clinically diagnosed case of TB involving organs other than the lungs (e.g. pleura, peripheral lymph nodes, abdomen, genitourinary tract, skin, joints and bones, meninges) (11). Musculoskeletal TB accounts for 1–2 % of all cases of TB out of which roughly 50 % of cases involve spinal column (12). 

 Mycobacterium tuberculosis is a rare cause of pyomyositis, accounting for 1.8 % of cases (13, 14).

 Disseminated tuberculous pyomyositis is an exceptionally rare extra pulmonary manifestation of TB, previously reported with a prevalence of 1.8% in a cohort study by Wang et al. (5). It is typically seen in immunocompromised individuals, although cases in immunocompetent patients have been documented (1, 3, 7). Clinical presentation is often nonspecific, including fever, weight loss, and localized pain or swelling, leading to delayed diagnosis.

 PET/CT plays a crucial role in mapping disease extent, detecting metabolically active lesions, and may aid in pinpointing optimal biopsy sites (4, 8). In musculoskeletal TB, early integration of ^18^F-FDG PET/CT can improve diagnostic accuracy, guide treatment planning, and assess response to therapy (9, 10). Currently available standard imaging modalities available: Magnetic resonance imaging (MRI) is the most useful imaging modality for diagnosis of pyomyositis with high sensitivity and specificity. It most clearly demonstrates diffuse muscle inflammation as well as any subsequent abscess formation (15). Moreover, MRI can demonstrate the extent of disease and associated underlying conditions such as osteomyelitis and septic arthritis (16). 

 But FDG PET/CT can prove to be a sensitive modality for imaging such patients with reduced amount of time and delineate the extent of metabolically active disease. As has been reported by Androunikou et al. (17), in a case report of presentation of extrapulmonary tuberculosis in a paediatric patient with limited lung disease but extensive extrapulmonary involvement with intense FDG uptake in vertebrae, psoas and paraspinal muscles, gluteus, longissimus, iliac nodes, and nasal lesion, consistent with widespread tuberculous pathology, and helped identify the site of biopsy in the case.

 Our case demonstrates the incremental value of PET/CT in identifying widespread skeletal muscle and nodal involvement, which would likely have been underestimated with conventional imaging alone.
